# The endosomal sorting complex required for transport repairs the membrane to delay cell death

**DOI:** 10.3389/fonc.2022.1007446

**Published:** 2022-10-18

**Authors:** Ye Yang, Min Wang, Ying-Ying Zhang, Shu-Zhi Zhao, Song Gu

**Affiliations:** ^1^ Obstetrics and Gynecology Department, Shanghai General Hospital, Shanghai Jiao Tong University School of Medicine, Shanghai, China; ^2^ General Surgery Department, Shanghai General Hospital, Shanghai Jiao Tong University School of Medicine, Shanghai, China; ^3^ Respiratory and Critical Care Medicine Department, Shanghai General Hospital, Shanghai Jiao Tong University School of Medicine, Shanghai, China; ^4^ Department of Ophthalmology, Shanghai General Hospital (Shanghai First People’s Hospital), Shanghai Jiao Tong University School of Medicine, Shanghai, China; ^5^ National Clinical Research Center for Eye Diseases, Shanghai Key Laboratory of Ocular Fundus Diseases, Shanghai Engineering Center for Visual Science and Photomedicine, Shanghai Engineering Center for Precise Diagnosis and Treatment of Eye Diseases, Shanghai, China; ^6^ Trauma Center, Shanghai General Hospital, Shanghai Jiao Tong University School of Medicine, Shanghai, China

**Keywords:** the endosomal sorting complex required for transport (ESCRT), apoptosis, necroptosis, pyroptosis, ferroptosis, autophagy

## Abstract

The endosomal sorting complex required for transport (ESCRT) machinery plays a key role in the repair of damaged plasma membranes with puncta form and removes pores from the plasma membrane in regulated cell death, apoptosis, necroptosis, pyroptosis, ferroptosis, and autophagy. ESCRT-I overexpression and ESCRT-III-associated charged multivesicular body protein (CHMP) 4B participate in apoptosis, and the ESCRT-1 protein TSG 101 maintains low levels of ALIX and ALG-2 and prevents predisposition to apoptosis. The ESCRT-III components CHMP2A and CHMP4B are recruited to broken membrane bubble sites with the requirement of extracellular Ca^2+^, remove membrane vesicles from cells, and delay the time required for active MLKL to mediate necroptosis, thus preserving cell survival. CHMP4B disturbed pyroptosis by recruiting around the plasma membrane neck to remove the GSDMD pores and preserve plasma membrane integrity depending on Ca^2+^ influx. The accumulation of the ESCRT-III subunits CHMP5 and CHMP6 in the plasma membrane is increased by the classical ferroptosis activators erastin-1 and ras-selective lethal small molecule 3 (RSL3) upon cytosolic calcium influx and repairs the ferroptotic plasma membrane. ESCRT-III- and VPS4-induced macroautophagy, ESCRT-0-initiated microautophagy. ESCRT-I, ESCRT-II, ESCRT-III, ALIX, and VPS4A are recruited to damaged lysosomes and precede lysophagy, indicating that ESCRT is a potential target to overcome drug resistance during tumor therapy.

## Introduction

The cell plasma membrane (PM) made of glycerophospholipids separates the inner and outer parts of the cell. Under physiological conditions, it acts as a gatekeeper to protect cells from the environment (1). In pathological situations, it undergoes structural and functional changes, resulting in cell damage (2). According to the recommendations of the Nomenclature Committee on Cell Death, cell death can be divided into accidental cell death (ACD) and regulated cell death (RCD); the latter, also called “active” cell death, can be further classified into apoptotic and non-apoptotic cell death with different morphological, genetic, and biochemical characteristics (3). Non-apoptotic cell death, such as necroptosis (4), pyroptosis (5), and ferroptosis (6, 7), triggers various types of plasma membrane damage to suppress tumor growth and could be used in the treatment of cancer against apoptosis resistance. Apoptosis, necroptosis, and pyroptosis all feature a passive “suicide” form promoting molecular processes and form a few nanometers of plasma membrane pores, leading to a catastrophic bursting of the cell (8). A rise in cytosolic calcium (Ca^2+^) has been related to the execution of different cell death processes, including apoptosis, necroptosis, pyroptosis, and unregulated necrosis (9). Apoptosis involves fragmentation and margination of chromatin, as well as generation of apoptotic bodies and plasma membrane blebbing (10). Necroptosis has been demonstrated to involve swelling of cells and was shown to be associated with the generation of released plasma membrane broken pieces as vesicles. Necrotic cells lose the integrity of the plasma membrane and thereby release intracellular damage-associated molecular patterns. Necroptosis signaling can be switched from apoptosis through anti-caspase mechanisms such as genetic ablation of caspase-8 (11). Pyroptosis relies on the protein gasdermin D (GSDMD) to precede rapid necrotic cell death by intensive blebbing. However, such features have not been observed during ferroptosis. Correspondingly, ferroptosis damages the active cell integrity with the accumulation of lipid peroxides due to a defective antioxidant response of the cell (12).

However, cells undergoing necroptosis do not always die. The known repair mechanisms involving the endosomal sorting complex required for transport (ESCRT) machinery outward vesiculates or sheds damaged membranes (13) and plays a critical counterbalancing role in sorting and downregulating activated cell-surface receptors (14) and repairing damaged plasma membranes to maintain membrane integrity (15), thus delaying cell death pathways, including apoptosis (16), necroptosis (17), pyroptosis (18, 19), ferroptosis (20), autophagy (21), and endosomal processing (22). In this review, we summarized the relationship between the ESCRT pathway and regulated cell death, aiming to identify ESCRT as a potential target to overcome drug resistance during tumor therapy.

### Summary of ESCRT function in cell death

ESCRT components are implicated in cellular events such as viral budding, cell signaling regulation (23), modulation of cytokine release, communication with immune cells (24), vesicle budding, and sustained cell viability and survival (25, 26). Four different multimeric protein complexes comprise ESCRT-0 to ESCRT-III machinery sequentially recruited from the cytoplasm to the endosomal membrane: ESCRT-0 (Vps27), ESCRT-I (Vps23, Vps28, Vps37, MVB12), ESCRT-II (Vps22, Vps25, Vps36), and ESCRT-III (Did2, Vps2, Vps20, Vps24, Snf7, Vps60, Chm7, Ist1). Another ESCRT machinery-functioning subcomplex, the AAA-ATPase complex vacuolar protein sorting-associated 4 (VPS4) and apoptosis-linked gene 2 (ALG-2)–interacting protein X (ALIX) protein, are potential decisive factors in the function of ESCRT-I and ESCRT-II. The ESCRT machinery mediates the “reverse-topology” cellular membrane scission mechanism (27) to process membrane remodeling, including cytokinesis, plasma membrane and lysosomal membrane repair, and nuclear envelope reformation (22). ESCRT-0 triggered by Ca^2+^ influx recruits other ESCRT machinery to endosomes during multivesicular body formation (MVB) sorting. Then, ESCRT-I binds ubiquitinated cargo and canonically activates ESCRT-II recruitment. ESCRT-III assembly starts with Vps20, followed by the core polymer subunits Vps2 and Vps24 binding Snf7 in tandem and finally recruiting VPS4, which dissociates the ESCRT structure from the endosomal membrane (28–31). Subsequently, the assembly of ESCRT-III machinery was recruited to wounds, assembled on the inner surface of the membrane neck to form single- or multiple-stranded polymorphic filaments shapes from spirals (32, 33) to conical spirals (34) and tubular helices (35), and then broken down within the lysosomal lumen to create plasma membrane blebs and intraluminal vesicles (ILVs) (36), mediate membrane scission, remove damaged parts of cell membranes away from the cytoplasm (27), and shed off the extracellular space.

ESCRT-I, ESCRT-II, ESCRT-III, VPS4A, and ALIX were recruited to damaged lysosomes and mediated lysosomal membrane repair. In mammalian cells, the ESCRT-III complex consists of 12 subunits: charged multivesicular body protein 1A (CHMP1A), CHMP1B, CHMP2A, CHMP2B, CHMP3, CHMP4A, CHMP4B, CHMP4C, CHMP5, CHMP6, CHMP7, and increased sodium tolerance 1 (IST1) (37). ESCRT-III plays a key role in the repair of damaged plasma membranes in various types of regulated cell death, such as necroptosis, pyroptosis, and ferroptosis. The inhibition of ESCRT-III machinery through genetic depletion of its core components increases susceptibility to anticancer agent-induced cell death (1), indicating that ESCRT III is a potential target to overcome drug resistance during tumor therapy.

### Apoptosis

Apoptosis is the most extensively investigated type of regulated cell death characterized by the sequential activation of cysteine-aspartic protease caspases (12). It is composed of the extrinsic and intrinsic major pathways that activate and cleave the downstream “executioner” caspase-3 and “initiator” caspase-7. Bcl-2 family effector proteins specific effector molecules B-cell/CLL lymphoma 2 (BCL2)-associated protein X (Bax) aBak and Bok (19, 38) regulated the integrity of the outer mitochondrial membrane permeabilization, releasing proteins of the mitochondrial intermembrane space into the cytosol. The intrinsic endolysosomal pathway in cellular stress (39) is enriched in phagosomes, and exosomes dominate the control of membrane budding and scission (36) to control apoptosis (40) in response to DNA damage (41). The extrinsic pathway is activated upon the binding of extracellular ligands to cell surface death receptors.

The ESCRT machinery components apoptosis-linked gene-2 (ALG-2) and exosomes interact with apoptosis-linked gene interacting protein X (ALIX) and are suggested to be a bridge between the endolysosomal system and apoptosis. ALG-2 is a penta-EF-hand protein enriched in phagosomes, also known as programmed cell death 6–interacting protein (PDCD6IP), which is regarded as apoptotic machinery in T-cell lines (42) and participates in T-cell receptor-, Fas-, and glucocorticoid-induced programmed cell death. ALIX interacts with Cbl-interacting protein of 85 kDa (CIN85)/SRC homology 3 (SH3) and sensitizes astrocytes to apoptosis in response to DNA damage (43, 44). The ALIX–ALG-2 complex undergoes apoptosis in a Ca2+/K+-dependent manner (45), while these events individually do not activate the downstream caspase cascade to eventually lead to apoptosis.

Tumor susceptibility gene 101 (TSG101) is an ESCRT-1 protein homologue of the yeast class E VPS protein complex ESCRT-III (46) that directly participates in mitigating ER stress-mediated apoptosis. The association of TSG 101 with ALIX prevents predisposition to apoptosis, but deregulating cytosolic Ca^2+^ and upregulating the levels of ALG-2 could disrupt this process. In healthy cells, when cytosolic Ca^2+^ is low, mahogunin RING finger 1 (MGRN1)-mediated ubiquitination of the ESCRT-I protein tumor susceptibility gene 101 (TSG101) promotes amphisomal–lysosomal and endolysosomal degradation pathways (47, 48) and helps maintain low levels of ALIX and ALG-2 as well as cell viability. MGRN1 depletion leads to cell surface glycoprotein mammalian PrP (^Ctm^PrP)-mediated ER stress, and an increase in cytosolic Ca^2+^ results in the ALIX–ALG-2 protein interaction. Overexpression of TSG101 also increases ALIX and ALG-2 levels, eventually eliciting predisposition to death in selected brain regions or myocardial apoptosis during embryonic development (16).

**Figure 1:**
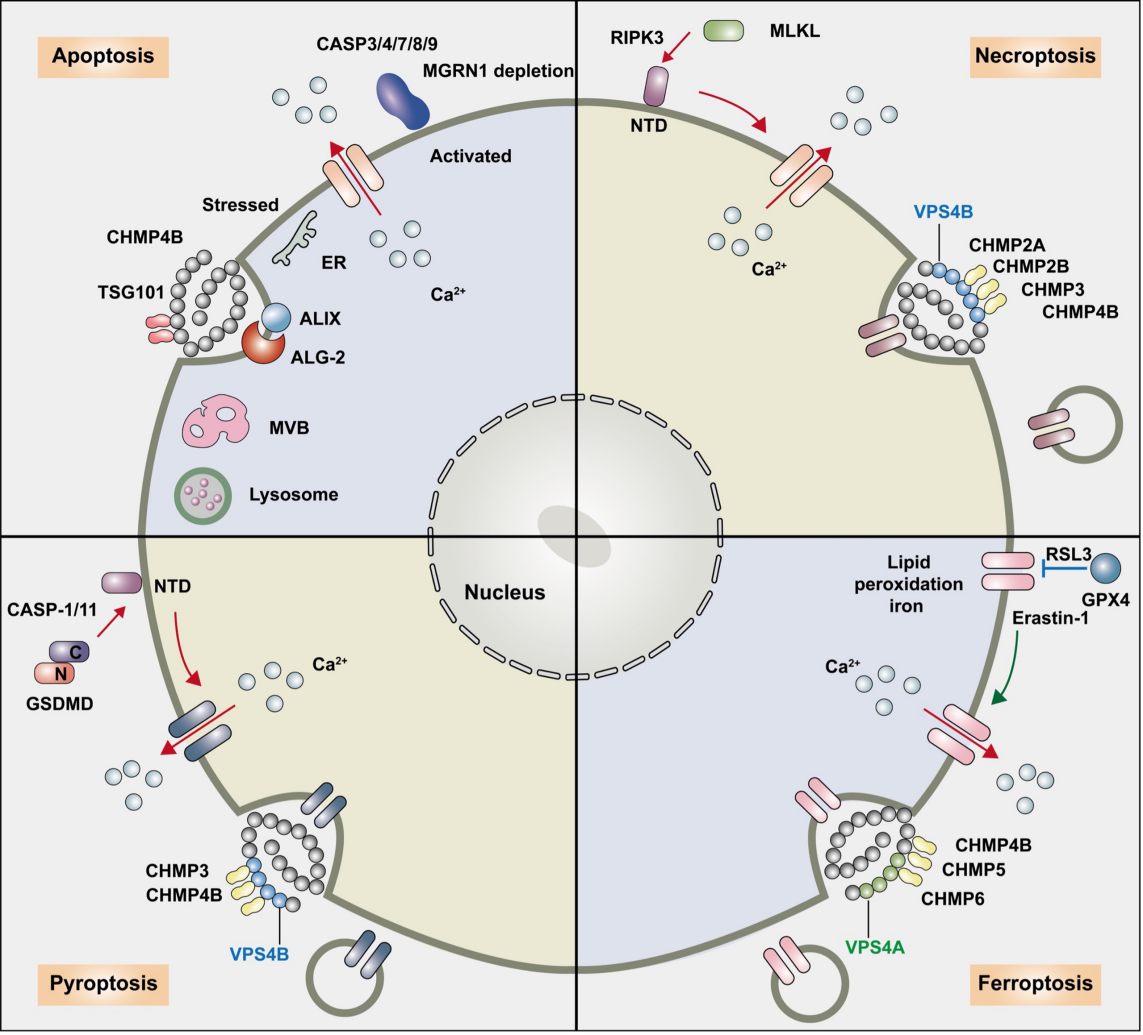
The relationship between the ESCRT pathway and regulated cell death. The endosomal sorting complex required for transport (ESCRT) machinery participates in the following regulated cell death: apoptosis, necroptosis, pyroptosis, ferroptosis and autophagy. ESCRT-I overexpression and ESCRT-III-associated charged multivesicular body protein (CHMP) 4B participate in apoptosis, and ESCRT-1 protein TSG 101 maintains low levels of ALIX and ALG-2 and prevents predisposition to apoptosis. The ESCRT-III components CHMP2A and CHMP4B are recruited to broken membrane bubble sites with the requirement of extracellular Ca2+, remove membrane vesicles from cells, and delay the time required for active MLKL to mediate necroptosis, thus preserving cell survival. CHMP4B disturbed pyroptosis by recruiting around the plasma membrane neck to remove the GSDMD pores and preserve plasma membrane integrity depending on Ca2+ influx. The accumulation of the ESCRT-III subunits CHMP5 and CHMP6 in the plasma membrane is increased by the classical ferroptosis activators erastin-1 and ras-selective lethal small molecule 3 (RSL3) upon cytosolic calcium influx and repairs the ferroptotic plasma membrane.

Under pathological conditions, the ESCRT-III-associated protein CHMP4B could also interact with ALIX-ALG-2 and participate in the endolysosomal system or cell apoptosis. CHMP4B mutation prevented early programmed cell death caused by overexpression of ALIX (49). However, increasing CHMP4B levels were accompanied by the upregulation of Fas receptor (Fas), Fas ligand (FasL), active caspase-8, and caspase-3 in neurons, which implicated a proapoptotic function in neuronal cells induced by hemin stimulation following intracerebral hemorrhage (ICH) *via* the extrinsic apoptotic pathway (50) (**Figure 1** and **Table 1**).

**Table 1:** The endosomal sorting complex required for transport (ESCRT) machinery plays a key role in the repair of damaged plasma membranes in regulated cell death, apoptosis, necroptosis, pyroptosis, ferroptosis, and autophagy.

	Gene ID	Full name	Gene description in NIH	Function	References
Apoptosis					
CHMP4B	128866	Charged multivesicular body protein 4B	Part of the endosomal sorting complex required for transport complex III (ESCRT-III)	Interact with ALIX-ALG-2, upregulation of Fas receptor (Fas), Fas ligand (FasL), active caspase-8, and caspase-3	(49, 50)
ALG-2	85365	Apoptosis-linked gene-2	A calcium-binding protein belonging to the penta-EF-hand protein family. Also known as programmed cell death 6 (PDCD6)	Apoptotic machinery in T-cell line.	(42)
ALIX	10015	Apoptosis-linked gene interacting protein X	Encodes a protein that functions within the ESCRT pathway.	ALG-2-ALIX complex is a bridge between the endolysosomal system and apoptosis	(43)
TSG101	7251	Tumor susceptibility gene 101	ESCRT-1 component, directly participates in mitigating ER stress-mediated apoptosis	Maintain low levels of ALIX and ALG-2, associated with ALIX prevents predisposition to apoptosis,	(46)
MGRN1	23295	Mahogunin ring finger 1	A C3HC4 RING-containing protein with E3 ubiquitin ligase activity *in vitro*	Mediated ubiquitination of the TSG101, MGRN1 depletion leads to cell surface glycoprotein mammalian PrP (^Ctm^PrP)-mediated ER stress	(16, 47, 48)
Necroptosis					
MLKL	197259	Mixed lineage kinase domain-like pseudokinase	Plays a critical role in tumor necrosis factor (TNF)-induced necroptosis, *via* interaction with key signaling molecule receptor-interacting protein 3 (RIP3)	ESCRT-III delayed the time required for active MLKL to disrupt plasma membrane integrity	(24)
FOXO1	2308	Forkhead transcription factor O1	Belongs to the forkhead family of transcription factors, binding to the promoter region	Resulting in decreased expression of RIP3 and p-MLKL and enhanced CHMP4B alleviates necroptosis	(51)
RIPK1	8737	Receptor-interacting kinase-1	Encodes a member of the receptor-interacting protein (RIP) family of serine/threonine protein kinases,	Mediated MLKL, plays a role in inflammation cell death necroptosis.	(52)
CHMP2A, CHMP2B CHMP3 CHMP4B				Remove phospho-MLKL–containing membrane vesicles from cells and block necroptosis	(17, 25, 53)
VPS4B	9525	Vacuolar protein sorting 4 Homolog B	a member of the AAA protein family (ATPases associated with diverse cellular activities), and is the homolog of the yeast Vps4 protein	promoting ALIX-syntenin 1-mediated scission and for association with MLKL to delay necroptotic cell death	(53)
**Pyroptosis**					
GSDMD	79792	Gasdermin D	a member of the gasdermin family, act as a tumor suppressor.	CHMP4B is recruited to the plasma membrane and clusters around the neck to remove the GSDMD pores and preserve plasma membrane integrity	(19)
VPS4B	9525	Vacuolar protein sorting 4 homolog B	a member of the AAA protein family	activated to dismantle the ESCRT-III complex after membrane scission	(54)
CHMP3				Knockdown of CHMP3 enhances GSDMD-mediated pyroptosis.	(54)
**Ferroptosis**					
GPX4	2879	Glutathione peroxidase 4	Belongs to the glutathione peroxidase family, members of which catalyze the reduction of hydrogen peroxide, organic hydroperoxides and lipid hydroperoxides, protect cells against oxidative damage	Best characterized trigger of ferroptosis	(55)
RSL3	123086821	ras-selective lethal small molecule 3	classical ferroptosis activators	Inactivate GPX4 to initiate ferroptosis	(52)
GSH	23687505	g-L-Glutamyl-L-cysteinylglycine		Inhibits classical ferroptosis activators erastin-1 causes GSH depletion	(56)
ESCRT-III: CHMP4B CHMP5/ CHMP6			Regulates membrane budding, fission	Repair and blocks erastin- and RSL3-induced ferroptotic cancer cell death	(20)
**Autophagy**					
**Macroautophagy**					
ATG 5	9474	Autophagy-related 5	In combination with autophagy protein 12, functions as an E1-like activating enzyme in a ubiquitin-like conjugating system	ATG 12-ATG 5 is needed under starvation conditions	(57)
ATG 12	9140	Autophagy-related 12	A component of ubiquitination-like systems involved in autophagy	ATG 12-ATG 5 is needed under starvation conditions	(58)
ATG 3	64422	Autophagy-related 3	A component of ubiquitination-like systems involved in autophagy	ATG 12-ATG 3 is required under nutrient-rich conditions	(58)
ATG8	852200	Autophagy-related 8	Involved in autophagy of the nucleus in macroautophagy	ATG8/MAP1LC3-I/II is a key player in autophagosomal extension	(59)
MAP1LC3	84557	Microtubule-associated protein 1 light chain 3	Microtubule-associated proteins which mediate the physical interactions between microtubules and components of the cytoskeleton	ATG8/MAP1LC3-I/II is a key player in autophagosomal extension	(59)
TSG101	7251	Tumor susceptibility gene 101	ESCRT-I subunit		(60)
VPS28	51160	AAA family ATPase VPS28	ESCRT-I subunit	Mediated a negative effect on autophagosome closure and autophagic substrate degradation	(60)
VPS4	856303	AAA family ATPase VPS4	ESCRT component, enables ATP binding and hydrolysis activity.	Responsible for OAM and IAM abscission and closure to form functional autolysosomes	(61)
CHMP2A	27243	Charged multivesicular body protein 2A	ESCRT-III component, recruited to the double-membrane autophagosome and mediates “reverse-topology” membrane fission	CHMP2A depletion or mutation induces ATG5- and LC3-II-positive phagophore accumulation, redirects protumorigenic autophagy to apoptosis	(61, 62)
CHMP2B	25978	Charged multivesicular body protein 2B	ESCRT-III component, functions in the recycling or degradation of cell surface receptors	CHMP2B mutant in primary neurons also leads to autophagosome and multilamellar body accumulation, resulting in neuronal cell loss	(63)
CHMP3	51652	Charged multivesicular body protein 3	ESCRT-III component, sorts transmembrane proteins into lysosomes/vacuoles *via* the multivesicular body (MVB) pathway	CHMP2A capping the assembled inner surface of the membrane neck with CHMP3 to drive membrane scission from the cytoplasm	(61)
CHMP4B	128866	Charged multivesicular body protein 4B	ESCRT-III component, functions in the sorting of endocytosed cell-surface receptors into multivesicular endosomes	Associated with the ALIX–ALG-2 complex was recruited to promote endosome maturation, permitting subsequent fusion between autophagosomes and MVBs to modulate the membrane	(64–66)
VPS32	2542922	AAA family ATPase VPS32	ESCRT III complex subunit	Snf7/Vps32 inactivation leads to the accumulation of autophagosomes	(60)
CEP55	55165	Centrosomal protein 55	Enables identical protein binding activity	CHMP2A, CHMP2B, CHMP3, CHMP7, and CEP55, VPS4 acted as potential regulators of phagophore closure, dissociated from the autophagic membrane, participated in nuclear envelope reformation	(67–69)
**Microautophagy**					
TORC	946252	Cytochrome c menaquinol dehydrogenase TorC	Endosomal target of rapamycin signaling complexes, a pentahemic c-type cytochrome that is anchored to the inner membrane	Directly control ESCRT components to inhibit untimely autophagy events	(70)
VPS27	855739	AAA family ATPase VPS27	The ESCRT-0 subunit	Relocated to the vacuolar membrane after a diauxic shift upon glucose starvation and was recruited less efficiently to the vacuolar membrane	(71)
myosin VI	4646	MYO6, myosin VI	Encodes a reverse-direction motor protein that moves toward the minus end of actin filaments and plays a role in intracellular vesicle and organelle transport	directly interacts with the ESCRT-0 Tom1 protein	(72, 73)
HSPA1A	3303	Heat shock protein family A (Hsp70) member 1A	Encodes a 70-kDa heat shock protein which is a member of the heat shock protein 70 family	Essential for regulating ESCRT-0/signal transducing adaptor molecule 2 (STAM2) and protecting cells from cytotoxicity by blocking ESCRT-0-initiated autophagosome–lysosome fusion	(74)
STAM2	10254	Signal transducing adaptor molecule 2	Closely related to STAM, an adaptor protein involved in the downstream signaling of cytokine receptors, both of which contain an SH3 domain and the immunoreceptor tyrosine-based activation motif (ITAM)	Protecting cells from cytotoxicity by blocking ESCRT-0-initiated autophagosome–lysosome fusion	(75)
**Lysophagy**					
ESCRT-I, ESCRT-II, ESCRT-III, ALIX, VPS4A				Recruited to damaged lysosomes and precede lysophagy	(76, 77)
TSG101 CHMP4B CHMP2A				TSG101 depletion inhibits CHMP4B recruitment to damaged lysosomes, whereas CHMP2A knockdown stabilizes it	(78, 79)

NIH, National Library of Medicine, National Center for Biotechnology Information Gene.

### Necroptosis

Necroptosis is characterized by permeability and finally plasma membrane rupturing in necrotic cells, different from shrinking and blebbing of plasma membranes in apoptotic cells (4, 19). Phosphatidylserine (PS) is localized to the inner leaflet of the plasma membrane of healthy cells, and exposure revealed small plasma membrane (PM) “bubbles” of broken plasma membrane released from the cell surface. Phospholipid scrambling (exposing PS) and disrupting plasma membrane integrity dominated the initial extracellular death signals and initiated necroptosis. Necroptosis can be mediated by tumor necrosis factor alpha (TNFα) and/or Fas or activated by the execution of necroptosis receptor-interacting kinase-1 (RIPK1)/RIPK3-mediated phosphorylation of mixed lineage kinase domains such as pseudokinase (MLKL) (24, 52). MLKL plays a critical role in tumor necrosis factor (TNF)-induced necroptosis *via* interaction with receptor-interacting protein 3 (RIP3). MLKL oligomerization-mediated PM disruption occurs prior to the loss of PM integrity, resulting in a rapid Ca^2+^ influx into the cell (17, 80) and induced bubbles, thus making the core machinery of the unique “membrane-explosive” necroptosis cell death pathway (80).

However, cells that are exposed to PS upon MLKL activation can be “resuscitated” and survive. The calcium-dependent ESCRT-III machinery plays a wider role in modulating various types of RCD by delaying cell membrane rupture. Sustained viability by ESCRT can either antagonize (pyroptosis) or enhance (necroptosis) the release of signaling events upstream of terminal effector activation (81). ESCRT-III greatly delayed the time required for active MLKL to disrupt plasma membrane integrity and the onset of membrane permeabilization, sustained the integrity of the plasma membrane, and enhanced necroptosis bubble formation, therefore sustaining survival of the cell (17, 82). When MLKL activation is subsequently halted, cells have sufficient time to permit surrounding cells to activate intracellular signaling pathways such as the cytokines C–X–C motif chemokine ligand 1 (CXCL1) and CXCL10 (80). Previous studies have indicated that the ESCRT pathway can remove phospho-MLKL–containing membrane vesicles from cells and block necroptosis, and CHMP4B decreases necroptosis through different transcriptional activators (24, 83). MLKL localizes to sites of broken membrane bubbles with the requirement of extracellular Ca^2+^, and ESCRT-III components CHMP2A or CHMP4B are then recruited and reduce cell membrane damage caused by p-MLKL, thus preserving survival despite MLKL activation in kidney transplantation (17, 25). The presence of the N-terminal ubiquitin-binding UEV domain in TSG101 and the disassembly complex VPS4B is critical in promoting ALIX-syntenin 1-mediated scission and for association with MLKL to delay necroptotic cell death (84). Forkhead transcription factor O1 (FOXO1) binds to the specific region on the CHMP4B promoter, and enhanced CHMP4B alleviates necroptosis in microglia by binding to the promoter region, resulting in decreased expression of RIP3 and p-MLKL and protecting against cell death after traumatic brain injury (TBI), thus improving neurological function recovery (51). On the other hand, the activation of necroptosis might alter the expression levels of ESCRT III proteins as a compensatory mechanism. CHMP2B is a marker for granulovacuolar degeneration (GVD) bodies in the Alzheimer’s disease (AD) brain (85). A significantly increased expression of CHMP2B, CHMP3, and VPS4B was shown in pMLKL+ neurons and counterbalanced necroptosis (53). CHMP2B mutation was associated with neurodegeneration in frontotemporal dementia (FTD) and amyotrophic lateral sclerosis (ALS) (86) (**Figure 1** and **Table 1**).

### Pyroptosis

Pyroptosis is a form of regulated necrosis induced by the pore-forming protein gasdermin D (GSDMD) that damages the plasma membrane (19). Caspase-1, caspase-4, caspase-5, and caspase-11 cleave GSDMD, release the N-terminal domain, and then translocate into the plasma membrane. While components of ESCRT-III mainly repaired the plasma membrane damage and function in preserving cell survival when the activity of the effectors is sufficiently low or the engaged pathway is disrupted prior to lysis (87). During pyroptosis, after cytosolic caspases cleave GSDMD to form nanoscale (10–15-nm) membrane pores, CHMP4B is recruited to the plasma membrane and clusters around the neck to remove the GSDMD pores and preserve plasma membrane integrity, thus limiting proinflammatory cytokine interleukin-1β (IL-1β) and IL-18 release through GSDMD pores (19), which rely on the influx of Ca^2+^. In contrast, VPS4B ATPase is activated to dismantle the ESCRT-III complex after membrane scission. Knockdown of CHMP3 enhances GSDMD-mediated pyroptosis. Recently, Kai et al. revealed that Ca^2+^ and K+ influx as well as activation of NLR family pyrin domain containing 3 (NLRP3)-dependent IL-1β release resulted in pyroptosis, and Mycobacterium tuberculosis (Mtb) infection spread to neighboring cells. Upon NLRP3 inflammasome activation, ESCRT composite ALG-2 and ALIX recruitment repaired plasma membrane damage in macrophages (54) (**Figure 1** and **Table 1**).

### Ferroptosis

Ferroptosis is a caspase-independent form of regulated pathological necroinflammation (56) and activation of the innate immune system, causing cell metabolic state changes involving cell enlargement, organelle swelling, membrane rupture, mitochondrial shrinkage, and increased outer membrane density (52, 88). Ferroptosis proceeds even in the absence of key effectors of apoptosis (Bax, Bak, and caspases) or necroptosis (MLKL, RIPK1, and RIPK3) (89). Cells dying by ferroptosis primarily exhibit shrunken and damaged mitochondria by electron microscopy, with few other morphological changes evident prior to the point of cell death (90, 91). The ferroptosis pathway occurs in cells involving targeting the amino acid antiporter system xc– or iron transport molecule shuttles such as transferrin (92) and lactotransferrin (93) or is activated after intracellular antioxidant enzymes are blocked (94). Glutathione peroxidase 4 (GPX4) is the best-characterized trigger of ferroptosis. Ferroptosis only occurs when the function of GPX4 is inactivated by ras-selective lethal small molecule 3 (RSL3) (55) or when it inhibits erastin-1, which causes g-L-glutamyl-L-cysteinylglycine (GSH) depletion (52, 56). Then, iron-dependent membrane phospholipid hydroperoxide accumulation precedes a sustained increase in cytosolic Ca^2+^ (95, 96), ultimately forming nanopores to trigger plasma membrane rupture and release of intracellular components (97).

ESCRT-III-dependent membrane repair blocks ferroptosis through association with the plasma membrane model and acts as a protective mechanism (20). The accumulation of the ESCRT-III subunits CHMP5 and CHMP6 in the plasma membrane is increased by the classical ferroptosis activators erastin-1 and RSL3, and the increase in cytosolic calcium influx relies on endoplasmic reticulum stress. CHMP5 or CHMP6 depletion increases erastin- and RSL3-induced ferroptosis (98). ESCRT-III is recruited to the plasma membrane to form CHMP4B puncta, removing pores from the plasma membrane, shedding them in ectosomes, and reducing lipid peroxidation as well as DAMP release, causing damaged membrane sections to be removed by endocytosis to delay ferroptosis membrane damage (81, 98–100). ESCRT-III also impacts cytokine secretion in ferroptotic cells (20). Apoptosis-inducing factor mitochondria-associated 2 (AIFM2)-dependent ESCRT-III recruitment regulates membrane budding, fission, and repair and blocks erastin- and RSL3-induced ferroptotic cancer cell death, which is responsible for ferroptosis resistance (101) (**Figure 1** and **Table 1**).

### Autophagy

Autophagy is a catabolic lysosomal degradation pathway responsible for nutrient recycling, protein and organelle quality control, and degradation and recycling of cellular material to maintain cell homeostasis and cope with stressful conditions (21). Autophagy is characterized by phagophores forming a small crescent-shaped membrane that stretches and seals cytoplasmic cargoes in double-membrane autophagosomes (21). Autophagosomes are nucleated from endoplasmic reticulum (ER) sites called omegasomes by phosphatidyl inositol 3 phosphate (PI3P) kinase complex class III and PI3P-binding proteins. Autophagosomes can fuse with early endosomes and MVBs to generate an intermediate compartment, the amphisome, which ultimately fuses with lysosomes (102). There are three most degradative systems types: the ubiquitin proteasome system, endocytosis, and autophagy vesicular processes converging on the lysosome (102). Lysosomes originate from endolysosomes or autolysosomes, act as single membrane-bound organelles, and recycle cellular nutrients through the outer membrane of mature autophagosomes and release acid hydrolases to degrade the autophagosomal content; they are also critical junctures between autophagy and endocytosis (103) as well as essential processes for maintaining intracellular homeostasis. ESCRT participates in four coexisting types of autophagy processes in a cell, namely, the recycling of cytosolic components by macroautophagy (often simply called autophagy), endosomal microautophagy, chaperone-mediated autophagy (CMA), and lysosomal and autophagic cell death pathways, ultimately directed to the lysosome for degradation (104). In macroautophagy, autophagic substrates are transported to the vacuole by autophagosomes with double-membrane structures; in microautophagy, substrates are directly engulfed by the vacuolar membrane.

### Macroautophagy

Macroautophagy is the main cytosolic degradative system involved in the formation of preautophagosomal structures called omegasomes (105). Autophagosome formation fuses with the lysosome to form an autolysosome, including extending, closing, and fusioning the isolation membrane (IM) dependent on autophagy-related (ATG) proteins to enwrap cargoes, thus initiating the macroautophagy degradation pathway characterized by double-membrane vesicles, termed autophagosome maturation, and encompassing multiple lysosomal-dependent mechanisms. The ESCRT machinery rescued slightly damaged lysosomes, and the ESCRT machinery and ATG proteins interact between endocytosis and macroautophagy to form a bridge between the endolysosomal system and cell death (82, 106). ATG12-ATG5 is needed under starvation conditions, while ATG12-ATG3 is required under nutrient-rich conditions (57, 58). Autophagosome closure requires a similar membrane scission machinery as ESCRT-III (107). ATG8/microtubule-associated protein 1 light chain 3 (MAP1LC3)-I/II is a key player in autophagosomal extension. Upon closure, LC3-II on the outer autophagosomal membrane (OAM) is delipidated and released to the cytosol, while LC3-II associated with the inner autophagosomal membrane (IAM) is degraded upon autophagosome–lysosome fusion (59).

Autophagosomal membranes can serve as activation platforms for intracellular death-inducing signaling complexes (iDISCs) to initiate caspase-8-dependent apoptosis. Mutations in ESCRT I (TSG101 and VPS28), II (SNF8, VPS22, VPS25), and III (VPS32) affected fusion between endosome and autophagosome accumulation to produce amphisomes (48, 60). The ESCRT-I subunit VPS28 variant mediated a negative effect on autophagosome closure and autophagic substrate degradation (108). The ESCRT component AAA-ATPase VPS4 is responsible for OAM and IAM abscission and closure to form functional autolysosomes (61) as well as dissociate the ESCRT machinery from the endosomal membrane (60). Prior to lysosomal recruitment, the autophagosome closure regulator ESCRT-III component CHMP2A is recruited to the double-membrane autophagosome and mediates “reverse-topology” membrane fission (61, 62), capping the assembled inner surface of the membrane neck with CHMP3 to drive membrane scission from the cytoplasm, shape MVB formation, and cut the membrane, and nuclear envelope reformation and remodeling processes involve regulating membrane fission phagophore closure (22, 61, 63, 109). CHMP2A depletion or mutation induces iDISC-mediated non-canonical caspase-8 activation on immature autophagosomal membranes and leads to ATG5- and LC3-II-positive phagophore accumulation, and redirects protumorigenic autophagy to apoptosis in osteosarcoma and neuroblastoma cells, thus inhibiting mouse xenograft model tumor growth (109–111), which may open new avenues for therapeutic targeting of autophagy in cancer.

**Figure 2:**
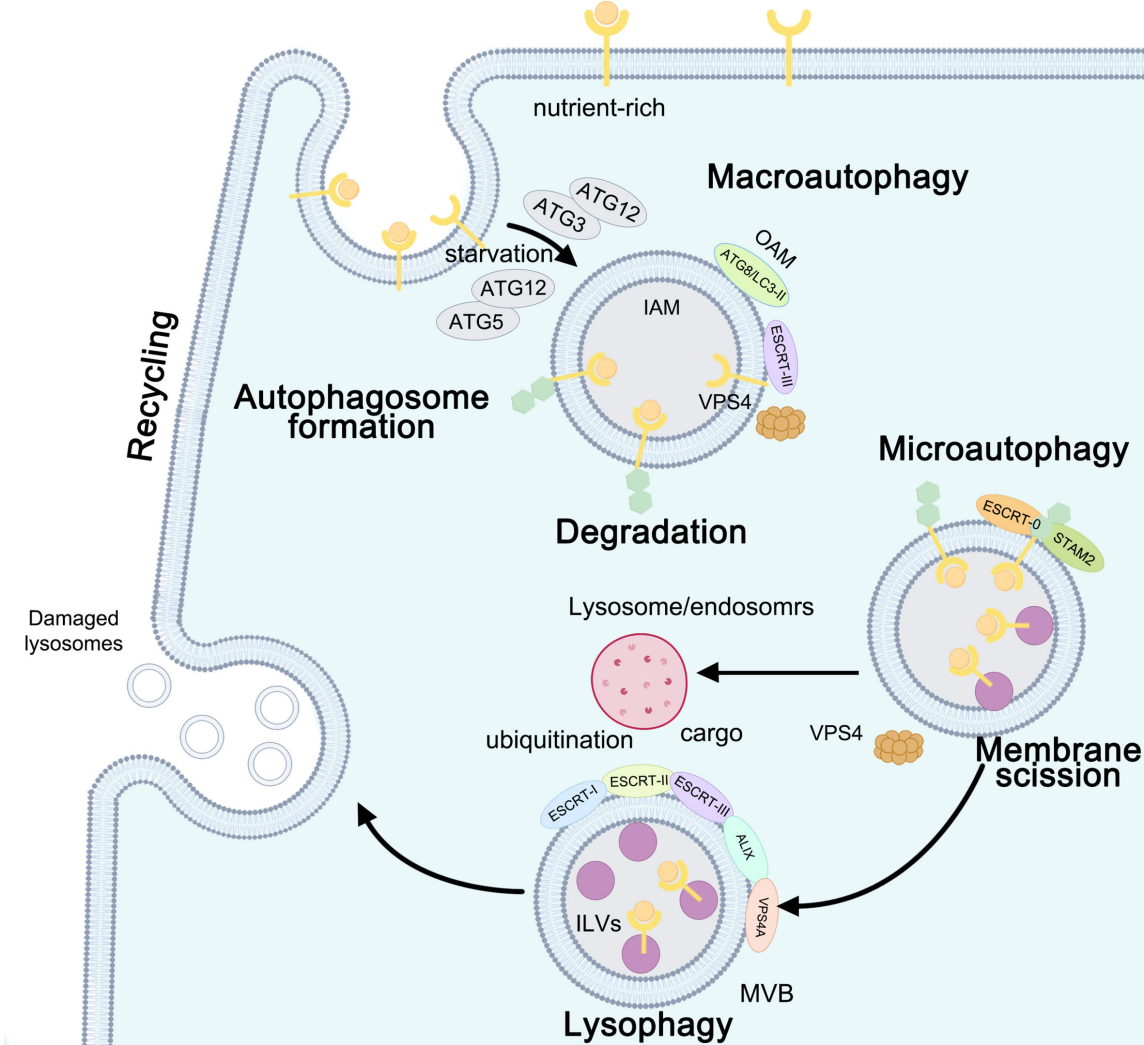
Autophagy: the function between ESCRT and macroautophagy, microautophagy, and lysophagy. ESCRT-III- and VPS4-induced macroautophagy is involved in the formation of autophagosomes: ATG12-ATG5 is needed under starvation conditions, while ATG12-ATG3 is required under nutrient-rich conditions. Upon closure, ATG8/light chain 3 (LC3)-II on the outer autophagosomal membrane (OAM) is delipidated and released to the cytosol, while LC3-II associated with the inner autophagosomal membrane (IAM) is degraded upon autophagosome-lysosome fusion. ESCRT-0- and signal transducingadaptor molecule 2 (STAM2)-initiated microautophagy requires membrane scission. ESCRT-I, ESCRT-II, ESCRT-III, ALIX, and VPS4A are recruited to damaged lysosomes and precede lysophagy.

Prior to final fusion with lysosomes, CHMP4B associated with the ALIX–ALG-2 complex was recruited to promote endosome maturation, permitting subsequent fusion between autophagosomes and MVBs to modulate the membrane (49, 64, 65, 112). ESCRT-III Snf7/Vps32 inactivation leads to the accumulation of autophagosomes, probably due to a blockage of autophagic flux in HeLa cells in the late stage of autophagosome formation (63, 66). Starvation-induced ESCRT-III components (CHMP2A, CHMP2B, CHMP3, CHMP7, and CEP55) as well as VPS4 acted as potential regulators of phagophore closure, dissociated from the autophagic membrane, participated in nuclear envelope reformation, and directly mediated membrane scission in human bone osteosarcoma epithelial cells (U-2 OS), HeLa cells, and human retinal pigment ephitilial-1 cells under basal and starved conditions (61, 67–69). The CHMP2B mutant in primary neurons also leads to autophagosome and multilamellar body accumulation, resulting in neuronal cell loss (63). The accumulation of autophagosomes in plant ESCRT mutants may result from inefficient closure of autophagosomes. The ESCRT-II subunit VPS36 localizes to endosomes and the plasma membrane. In Arabidopsis, autophagic turnover of plastids decreased in the ESCRT-related CHMP1 (VPS46) mutant due to defects in phagophore maturation and transport (113) (**Figure 2** and **Table 1**).

### Microautophagy

Microautophagy requires membrane scission at the vacuolar membrane, similar to ILV formation at the MVE. Microautophagy occurs in the following three different membrane dynamics: protrusion of the lysosomal membrane to engulf the cargo, invagination of the lysosomal membrane or endosome to entrap the cargo inside the lysosome or endosomes (114). The ESCRT machinery plays catabolic roles in cell starvation through the sorting and degradation of cytosolic proteins and lipids, similar to a hub-like system involved in the final maturation of both late endosomes and autophagosomes. ESCRT participates in plasma membrane scission as well as in cytosolic components, proteins, and lipids during starvation and affects the fusion of vesicles with lysosomes to form autolysosomes (115, 116). Upon ER stress-induced macrolipophagy in budding yeast, the whole ESCRT machinery is recruited to the scission site on the vacuolar membrane and lipid droplets to remove the ER whorls and Snf7, thus clearing the defective proteasomes (117). The ESCRT-0 subunit Vps27 relocated to the vacuolar membrane after a diauxic shift upon glucose starvation and was recruited less efficiently to the vacuolar membrane; endosomal target of rapamycin (TOR) signaling complexes (TORC) directly control ESCRT components to inhibit untimely autophagy events (70, 71). In mammalian cells, on the one hand, myosin VI directly interacts with the ESCRT-0 Tom1 protein; on the other hand, myosin VI interacts with autophagy adaptors and optineurin, which are involved in selective autophagy (72, 73). Heat shock protein family A (Hsp70) member 1A (HSPA1A) is essential for regulating ESCRT-0/signal transducing adaptor molecule 2 (STAM2) and protecting cells from cytotoxicity by blocking ESCRT-0-initiated autophagosome–lysosome fusion (74), abolishing autophagic flux in cellular thermoresistance, significantly reducing thermal cytotoxicity, and promoting cell survival (75) (**Figure 2** and **Table 1**).

### Lysophagy

Lysophagy occurs only when the repair mechanism fails to be initiated due to extensive damage to the lysosomal membrane and the inability to recruit ESCRT repair complexes. Damaged lysosomes are selectively eliminated by lysophagy when ESCRT-mediated membrane repair fails (118). Rescue of lysosomes mediated by ESCRT, removal of damaged lysosomes *via* lysophagy, and lysosome biogenesis can restore lysosome function and improve autophagic clearance (118). ESCRT-mediated membrane remodeling may contribute to both immediate and delayed responses to lysosomal damage by the multivesicular body pathway (76, 77). ESCRT-III is recruited to damaged lysosomes, which requires ESCRT-I and ALIX. ESCRT-I, ESCRT-II, ESCRT-III, ALIX, and VPS4A are recruited to damaged lysosomes and precede lysophagy. TSG101 depletion inhibits CHMP4B recruitment to damaged lysosomes, whereas CHMP2A knockdown stabilizes CHMP4B. ESCRT recruitment protects cells against cell death caused by lysosome damage (78, 79). After membrane damage, ubiquitination is delayed by 30 min compared to the recruitment of ESCRT components (77). Dampening ESCRT responses by depleting TSG101 and ALIX slows or completely blocks this rapid recovery and thereby implicates ESCRT function in lysosomal repair (119, 120) (**Figure 2** and **Table 1**).

### ESCRT as a potential target for tumor therapy

Tumor cells develop drug-resistance effects to escape cell death and cause treatment failure. ESCRT contributes to resistance to cell death and is generally regarded as a tumor-suppressor gene. The ESCRT-0 protein Hrs was upregulated in tumor specimens of the stomach, colon, liver, cervix, and melanoma (121). The ESCRT-I subunit Vps37A was significantly reduced in the hepatocellular carcinoma cell line (122). ESCRT-III subunit CHMP1A overexpression inhibited tumor xenograft growth of human pancreatic carcinoma cells (123). Upregulation of CHMP3 was detected in non-small cell lung carcinoma (124). CHMP5 or CHMP6 confers resistance to ferroptotic PANC1 and HepG2 human cancer cell death (98). CHMP4C^T232^ is associated with increased susceptibility to cancer tumorigenesis in ovarian cancer (125) and male genital tract, prostate, and skin cancers (126). Inhibition of these ESCRT proteins could block membrane remodeling and induce cancer cell death. In addition, observed from the uterine corpus endometrial carcinoma (UCEC) dataset of The Cancer Genome Atlas database (TCGA) database (https://portal.gdc.cancer.gov/) and GEPIA (http://gepia.cancer-pku.cn/), the ESCRT-III components CHMP2A, CHMP4B, CHMP4C, CHMP5, and CHMP6 were significantly related to tumor-infiltrating lymphocytes (TILs), revealing that a deregulated ESCRT pathway would offer a potential target or effective markers in cancer immunotherapy.

## Discussion

Apoptosis is a non-inflammatory form of PCD mediated by activation of apoptotic caspases and can occur either *via* an extrinsic or an intrinsic pathway that converges on the activation of the executioner caspase-3, 6, and 7 (12). At the terminal stage of apoptosis, cells are phagocytosed *in vivo* by scavenger cells, such as macrophages or neutrophils. However, if these cells are not removed in a timely fashion, as is the case *in vitro*, they progress to a final phase called secondary necrosis characterized by cytoplasmic swelling and plasma membrane damage, similar to the phenotype of cells undergoing pyroptosis or necroptosis (8, 9). Necroptosis is triggered by the activation of receptor-interacting protein kinase-3 (RIPK3), which phosphorylates the pseudokinase MLKL, causing it to translocate to the plasma membrane to induce cell permeabilization (56). Pyroptosis is triggered primarily by activation of inflammatory caspases, which include caspase-1 and caspase-11 (caspase-4/-5 in humans) (19). Autophagy is a process of bulk protein degradation in which cytoplasmic components, including organelles, are enclosed in double-membrane structures called autophagosomes and delivered to lysosomes or vacuoles for degradation (21). The ESCRT machinery is involved in the above regulated cell death processes, such as apoptosis, necroptosis, pyroptosis, ferroptosis, and autophagy, in a Ca^2+^-dependent manner (47, 48). Ca^2+^ influx plays an important role in the activation and recruitment of the ESCRT-III complex, leading to the repair of damaged plasma membranes during cell death. The ESCRT-1 protein TSG 101 maintains low levels of ALIX and ALG-2 and prevents predisposition to apoptosis (47, 48). ESCRT-III components CHMP2A or CHMP4B are recruited to the broken membrane bubble sites with the requirement of extracellular Ca^2+^, delaying the time required for active MLKL to mediate necroptosis and thus preserving cell survival despite MLKL (17, 25). Upon Ca^2+^ and K+ influx activating the NLRP3 inflammasome, the ESCRT composites ALG-2, ALIX, and CHMP4B are recruited around the plasma membrane neck to remove the GSDMD pores and block pyroptosis and thus preserve plasma membrane integrity (19, 54). The accumulation of the ESCRT-III subunits CHMP5 and CHMP6 in the plasma membrane is increased by the classical ferroptosis activators erastin-1 and ras-selective lethal small molecule 3 (RSL3) upon cytosolic calcium influx and repairs the ferroptotic plasma membrane (98). ESCRT is also involved in membrane scission machinery on autophagosome closure, and mutations in ESCRT I (TSG101 and VPS28), II (SNF8, VPS22, VPS25), and III (VPS32) affect fusion between endosome and autophagosome accumulation to produce amphisomes (48, 60).

## Conclusion

In summary, ESCRT provides time to the dying cell, and ESCRT-dependent membrane repair negatively regulates cell death: ESCRT-I and -III participate in apoptosis, ESCRT-III mediates necroptosis, pyroptosis, and ferroptosis, ESCRT-0 initiates microautophagy, ESCRT-III induces macroautophagy, and ESCRT-I, -II, and -III precede lysophagy. All these types of cell death can cause plasma membrane damage through different mechanisms; however, some essential mechanisms need to be clarified, such as the exact time point or target site ESCRT-rescued cell death, and it will be interesting to investigate the role of ESCRT as a potential target to overcome drug resistance in cancer cells.

## Author contributions

YY and MW analyzed and interpreted the data. Y-YZ and SG collected information. YY, MW, Y-YZ, S-ZZ and SG worked equally as major contributors in writing the manuscript. All authors contributed to the article and approved the submitted version.

## Funding

This work was supported by the National Natural Science Foundation of China (81902628, 81900036), Clinical Research Project of Shanghai Health Commission (202040455), and Shanghai Songjiang District Science and Technology Commission for funding the project (20SJKJGG139, 20SJKJGG304)

## Acknowledgments

We thank Elsevier Author Services (https://webshop.elsevier.com/language-editing-services/language-editing/) and for its linguistic assistance during the preparation of this manuscript. We thank American Journal Experts (AJE)(https://www.aje.com) for helping us revise the manuscript.

## Conflict of interest

The authors declare that the research was conducted in the absence of any commercial or financial relationships that could be construed as a potential conflict of interest.

## Publisher’s note

All claims expressed in this article are solely those of the authors and do not necessarily represent those of their affiliated organizations, or those of the publisher, the editors and the reviewers. Any product that may be evaluated in this article, or claim that may be made by its manufacturer, is not guaranteed or endorsed by the publisher.

## Glossary

**Table:**

ACD	accidental cell death
AD	Alzheimer’s disease
ALG-2	apoptosis-linked gene 2
ALIX	apoptosis-linked gene interacting protein X
ALS	amyotrophic lateral sclerosis
AIFM2	apoptosis-inducing factor mitochondria-associated 2
ATG	autophagy related
Bax	B-cell/CLL lymphoma 2 (BCL2)-associated protein X
Ca^2+^	calcium
CMA	chaperone-mediated autophagy
CHMP2A	charged multivesicular body protein 2A
CIN85	CbI-interacting protein of 85 kDa
CXCL1	C–X–C motif chemokine ligand 1
ER	endoplasmic reticulum
ESCRT	endosomal sorting complex required for transport
FTD	frontotemporal dementia
FOXO1	Forkhead transcription factor O1
GPX4	glutathione peroxidase 4
GSDMD	gasdermin D
GSDME	gasdermin E
GSH	g-L-glutamyl-L-cysteinylglycine
GVD	granulovacuolar degeneration
IAM	inner autophagosomal membrane
LAMP-2A	lysosome-associated membrane protein 2A
ICH	intracerebral hemorrhage
LC3	light chain 3
iDISCs	intracellular death-inducing signaling complexes
ILVs	intraluminal vesicles
IL-1β	interleukin-1β
IM	isolation membrane
IST1	increased sodium tolerance 1
FasL	Fas ligand
HSPA1A	heat shock protein family A (Hsp70) member 1A
MLKL	mixed lineage kinase domain-like pseudokinase
MGRN1	mice deficient in the RING domain–containing E3 ligase mahogunin RING finger 1
Mtb	Mycobacterium tuberculosis
MVB	multivesicular body formation
NVT	NBR1-mediated vacuolar targeting
NLRP3	NLR family, pyrin domain containing 3
OAM	outer autophagosomal membrane
PDCD6IP	programmed cell death 6–interacting protein
PM	plasma membrane
PS	phosphatidylserine
PI3P	phosphatidyl inositol 3 phosphate
RIPK3	receptor interacting serine/threonine kinase 3
RIP3	receptor-interacting protein 3
RCD	regulated cell death
RSL3	ras-selective lethal small molecule 3
STAM2	signal transducing adaptor molecule 2
SH3	SRC homology 3
TCGA	dataset of The Cancer Genome Atlas database
TBI	traumatic brain injury
TILs	tumor-infiltrating lymphocytes
TSG101	tumor susceptibility gene 101
TNFα	tumor necrosis factor alpha
TOR	target of rapamycin
UPS	ubiquitin/proteasome system
UCEC	uterine corpus endometrial carcinoma
VPS4	vacuolar protein sorting-associated 4
